# Supplementary material for: Mucins 3A and 3B Are Expressed in the Epithelium of Human Large Airway
Source: Int J Mol Sci. 2023 Aug 31;24(17):13546. doi: 10.3390/ijms241713546 (PMC10487631; doi:10.3390/ijms241713546)
Supplement: Supplementary file 1 [file ijms-24-13546-s001.zip › ijms-2521299-supplementary.pdf]

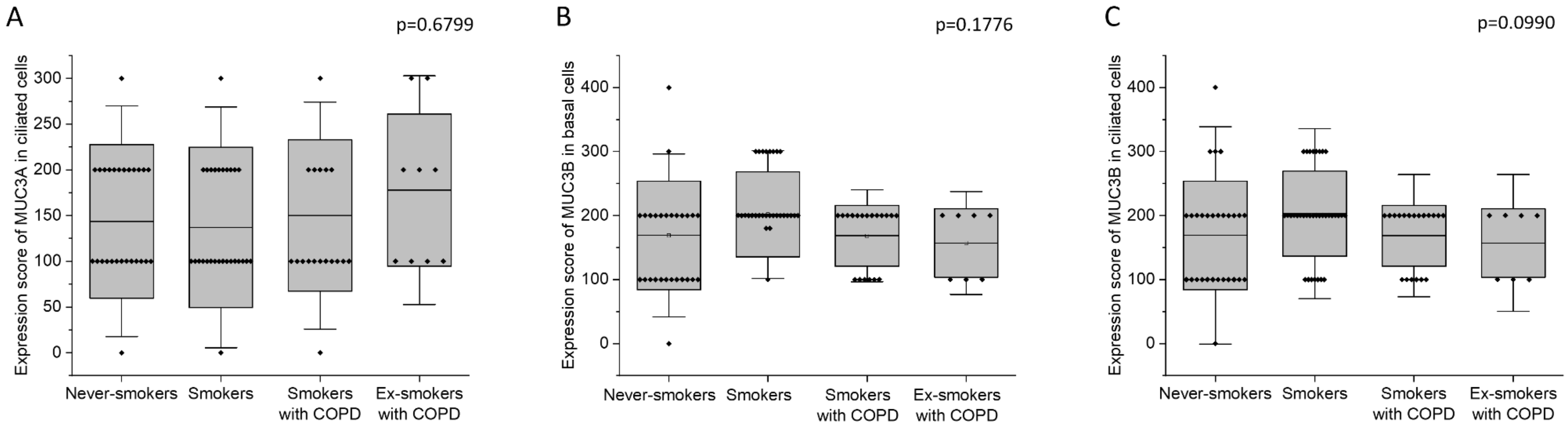


**Figure S1.** The immunohistochemical expression of MUC3A in ciliated cells (A), MUC3B in basal cells (B), and MUC3B in ciliated cells (C) across the four study groups. Kruskall-Wallis test in A-C. Bars represent mean and standard deviation.
